# Are Iron-Phosphate Minerals a Sink for Phosphorus in Anoxic Black Sea Sediments?

**DOI:** 10.1371/journal.pone.0101139

**Published:** 2014-07-02

**Authors:** Nikki Dijkstra, Peter Kraal, Marcel M. M. Kuypers, Bernhard Schnetger, Caroline P. Slomp

**Affiliations:** 1 Department of Earth Sciences - Geochemistry, Faculty of Geosciences, Utrecht University, Utrecht, The Netherlands; 2 Department of Biogeochemistry, Max Planck Institute for Marine Microbiology, Bremen, Germany; 3 Microbiogeochemistry, Institute for Chemistry and Biology of the Marine Environment (ICBM), University of Oldenburg, Oldenburg, Germany; Università della Calabria, Italy

## Abstract

Phosphorus (P) is a key nutrient for marine organisms. The only long-term removal pathway for P in the marine realm is burial in sediments. Iron (Fe) bound P accounts for a significant proportion of this burial at the global scale. In sediments underlying anoxic bottom waters, burial of Fe-bound P is generally assumed to be negligible because of reductive dissolution of Fe(III) (oxyhydr)oxides and release of the associated P. However, recent work suggests that Fe-bound P is an important burial phase in euxinic (i.e. anoxic and sulfidic) basin sediments in the Baltic Sea. In this study, we investigate the role of Fe-bound P as a potential sink for P in Black Sea sediments overlain by oxic and euxinic bottom waters. Sequential P extractions performed on sediments from six multicores along two shelf-to-basin transects provide evidence for the burial of Fe-bound P at all sites, including those in the euxinic deep basin. In the latter sediments, Fe-bound P accounts for more than 20% of the total sedimentary P pool. We suggest that this P is present in the form of reduced Fe-P minerals. We hypothesize that these minerals may be formed as inclusions in sulfur-disproportionating *Deltaproteobacteria*. Further research is required to elucidate the exact mineral form and formation mechanism of this P burial phase, as well as its role as a sink for P in sulfide-rich marine sediments.

## Introduction

Phosphorus (P) is a key nutrient for marine organisms and its availability may limit primary production, on both short and long timescales. Most P is efficiently recycled in the marine realm, but the long-term availability of P ultimately depends on the balance between inputs from rivers and burial in the sediment [1]–[3].

In most marine settings, organic P is the dominant form of P deposited at the sediment-water interface [4]. Part of this organic P is degraded in the sediment and dissolved phosphate is subsequently released to the bottom water or pore water (mainly HPO_4_^2−^ in seawater; henceforth termed PO_4_). In sediments overlain by oxic bottom waters, PO_4_ can be bound to iron (oxyhydr)oxides (henceforth termed Fe-oxide bound P) in the surface sediment. Upon burial or mixing of this Fe-oxide bound P into deeper anoxic sediments, PO_4_ may be released due to reductive dissolution of the Fe (oxyhydr)oxides. The released PO_4_ can precipitate in the form of authigenic carbonate fluorapatite (authigenic Ca-P) at depth, or diffuse upward where it can again be bound to Fe (oxyhydr)oxides in the oxic surface sediment [2], [5]. In marine sediments overlain by oxic bottom water, organic P, Fe-oxide bound P and authigenic Ca-P are typically the major sedimentary phases contributing to burial of P [2], [5].

Sediments that are overlain by anoxic and sulfidic (“euxinic”) bottom waters can contain organic P as the dominant P burial phase, as demonstrated recently for Baltic Sea sediments [6], [7]. In such sediments, dissimilatory Fe(III) reduction and dissolution of Fe(III) (oxyhydr)oxides by reaction with hydrogen sulfide (H_2_S) are generally expected to lead to low Fe-oxide bound P concentrations in the surface sediments. The lack of active Fe and P recycling in the sediment can limit the accumulation of pore water PO_4_ in the surface sediments and thus hamper the formation of authigenic Ca-P [2], [5], [8]. Other factors, such as the alkalinity and concentration of dissolved Ca^2+^ in the pore water and the potential for coprecipitation of P with Mn-carbonate, also play a role in authigenic Ca-P formation in anoxic sediments (e.g. [4], [9]).

Surprisingly, Jilbert and Slomp [9] recently observed high Fe-bound P contents in sediments from two near-permanently euxinic basins in the Baltic Sea (±15 µmol/g; ∼20% of the total P pool). This Fe-bound P was extracted in the step targeting P bound to reactive Fe (oxyhydr)oxides in the ‘SEDEX’ sequential extraction procedure developed by Ruttenberg [10]. Moreover, electron microprobe energy-dispersive spectroscopy allowed the identification of discrete Fe-P enrichments in these Baltic Sea sediments. All sample handling took place under strictly anoxic conditions, which rules out conversion of other sedimentary P phases to Fe-oxide bound P upon exposure to oxygen during sample handling as described by Kraal et al. [11].

The relatively large amount of P that was removed during the SEDEX step that targets Fe-oxide P may be explained by the fact that this step can also extract reduced Fe-P minerals such as vivianite (Fe(II)_3_(PO_4_)_2_·8H_2_O) [12]. Reduced Fe phosphates have been suggested to form below the sulfate/methane transition zone (SMTZ) in Zambezi deep-sea fan sediments [13] and in surface sediments in the Bothnian Sea [14]. In both studies, pore water Fe^2+^ is no longer scavenged by dissolved H_2_S below the SMTZ and is available for the formation of reduced Fe phosphates. In the study of Jilbert and Slomp [9], however, reduced Fe phosphates are suggested to form *within* the SMTZ where Fe^2+^ concentrations are low and pore waters are undersaturated with respect to vivianite. In these sediments, localized rapid reductive dissolution of Fe-oxides and the associated release of P might still enable precipitation of reduced Fe phosphates [9]. It thus appears that reduced Fe phosphates can form in anoxic marine sediments in a wide range of modern environmental settings. Reduced Fe phosphates may also have formed in subsurface waters in the geologic past during periods of anoxic, ferruginous conditions (e.g. [15]). The underlying processes and the general importance of the burial of reduced Fe phosphates in marine systems remain to be determined, however.

In this study, we analyzed the upper 30–50 cm of sediment from 6 stations in the western Black Sea to determine whether Fe-bound P is an important P pool in sediments that are permanently overlain by euxinic bottom waters [16]. In addition to geochemical analyses of the pore water, we determined the total elemental composition, organic C and N content, and the sediment forms of Fe and P using sequential chemical extractions. Our results show that sediments in the Black Sea contain a large pool of Fe-bound P, lending further support to its importance as a P sink in anoxic, sulfidic basins.

## Materials and Methods

### Ethics statement

All necessary permits were obtained to sample the study sites for scientific purposes during the R/V Meteor Cruise 51/4 and 72/5 (granted by the Romanian and Ukranian authorities).

### Study area, stratigraphy and coring sites

**Figure 1:**
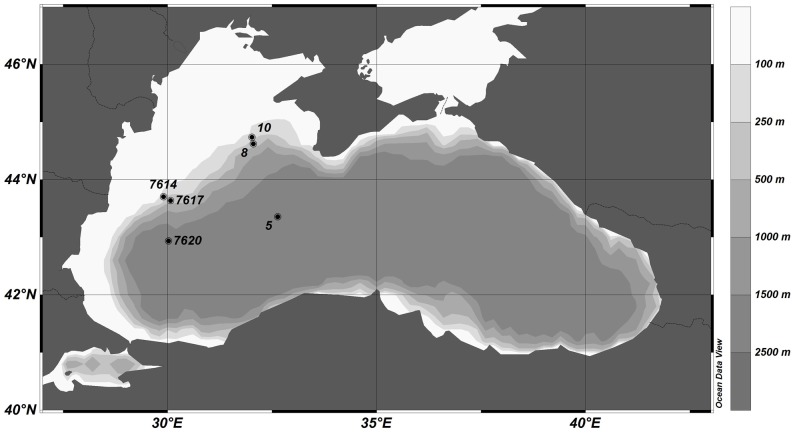
Black Sea map including the station locations and bathymetry in m below sea surface (mbss).

The Black Sea, which is a permanently euxinic marine basin, has a surface area of 423,000 km^2^ and a maximum water depth of 2200 m [17] (Fig. 1). Saline water is supplied from the Mediterranean Sea through the Bosporus Strait, and most fresh water enters the basin through the Danube and other large European rivers. This continuous supply of both fresh and saline water has led to a strong salinity stratification with highest salinities in the deep water, and a stable redoxcline at a depth of about 150 m [18], [19].

Two depositional periods can be discerned within the sedimentary profiles as captured by multicores: Unit II (∼7.5–2.5 kyr BP) and Unit I (∼2.5 kyr BP – present) [17]–[19]. Unit I sediments are characterized by the preservation of coccolithophores as finely laminated calcareous ooze containing 1–10% organic carbon. Unit II is also organic-rich but is devoid of the remains of coccolithophores. Turbidite deposits are common in deep basin sediments of the Black Sea. These homogeneous sediment layers originate from lateral transport from the shelf and slope areas towards the basin [20]. Shelf sediments are not characterized by distinct units and are overlain by oxic bottom water. They are mainly light brown/gray in color and contain shells [20].

In total, 6 multicores were retrieved from two transects in the western part of the Black Sea (Fig. 1). Cores from stations 7614, 7617 and 7620 were collected during the R/V Meteor Cruise 51/4 in December 2001. Stations 5, 8 and 10 were sampled during the R/V Meteor Cruise 72/5 in May and June 2007. Station 7614 is the only sample site that is situated on the continental shelf (<200 m water depth); all other stations are located in the basin (>200 m water depth, including the slope area). All sediment cores from the basin contained Unit I deposits (Table 1). Only cores from station 7620 and station 5 contained Unit II deposits, below 27 cm and 33 cm sediment depth, respectively. The core from station 5 also contained a homogeneous turbidite layer between 26 and 33 cm sediment depth (Table 1).

**Table 1:** Key characteristics of the sampled stations.

R/V Meteor Cruise & Year of sampling	Station	Water Depth(m)	Latitude	Longitude	*Unit l* (cm)	*Unit ll* (cm)	Turbidite (cm)	Bottom water redox-conditions
Cruise 51/4	7614	91	43°42.8′	29°54.4′	-	-	-	Oxic
2001	7617	464	43°38.9′	30°04.1′	<35			Anoxic
	7620	2055	42°56.2′	30°01.9′	<27	27–33		Anoxic
Cruise 72/5	10	459	44°44.5′	32°01.2′	<45			Anoxic
2007	8	1060	44°37.7′	32°03.2′	<36			Anoxic
	5	2105	43°21.8′	32°38.0′	<26,	33–35*	26–33	Anoxic

*: At station 5, the assignment of the interval from 33–35 cm to unit II is tentative and is based on visual observations.

### Bottom water and pore water analyses'

Bottom water and pore water data were only available for stations 5, 8 and 10. After bottom water samples were taken, the cores were sampled for pore water using rhizons (pore size: 0.1 µm) inserted through pre-drilled holes in the core liner [21] to determine dissolved Fe^2+^, manganese (Mn^2+^), PO_4_ and sulfate (SO_4_^2−^) concentrations. Subsamples were acidified with 1 M HNO_3_ and stored at 5°C before analysis by ICP-OES. H_2_S was assumed to be released during the initial acidification, thus total S is used as a measure for sulfate (SO_4_^2−^). Rhizons were also used to collect pore water from a replicate core to determine H_2_S (after zinc acetate fixation; [22]). At stations 5 and 10, pore water was collected from cores from parallel deployments with rhizons and subsampled at 9°C for the colorimetric determination of ammonium (NH_4_^+^) [23] and alkalinity [24].

### Total sediment composition

Multicores were sectioned on board and the sediment slices were placed into glass vials or sample bags and frozen at minus 20°C. On land, the frozen sediment slices were freeze-dried and ground. While the sediments of sites 7614, 7617 and 7620 were exposed to the atmosphere during sample handling, those of sites 5, 8 and 10 were always handled under an inert nitrogen (N2) or argon (Ar) atmosphere. Subsamples (0.125 g) of ground sediment slices were digested at 90°C in a mixture of hydrofluoric acid, nitric acid and perchloric acid, after which the acids were evaporated and the residue was redissolved in 1 M nitric acid. The final solution was then analyzed for total P, manganese (Mn), calcium (Ca), sulfur (S), Fe, aluminum (Al), and molybdenum (Mo) using inductively coupled plasma optical emission spectrometry (ICP-OES; Perkin Elmer Optima 3000). The relative error, based on analyses of laboratory reference material (ISE-921) and sample replicates, was generally less than 5%.

Calcium carbonate contents (CaCO_3_) were estimated from total Ca contents, after correcting for the Ca content in clays: CaCO3 = 2.5 x (Ca content - 0.345 x Al content), where 0.345 is the Ca/Al ratio in clays [25], [26]. Given that Black Sea sediments also may contain dolomite, CaCO3 contents may be overestimated. Organic C contents were determined using sediment subsamples (0.2 g) that were decalcified by two washes with 1 M hydrochloric acid (HCl; 4 and 12 hours, respectively) and a final rinse with UHQ water. The decalcified samples were freeze-dried and organic C was measured with a CN analyzer (Fisons Instruments NA 1500). Laboratory tests have shown that the amount of organic C hydrolyzed by 1 M HCl is negligible [27]. Based on laboratory reference materials and replicates, the relative error for organic C was generally less than 5%.

### Sediment phosphorus fractionation

Phosphorus fractionation in sediment subsamples was determined with the SEDEX method [10], as modified by Slomp et al. [5], but including the exchangeable P step. The first extraction (0.5 h) with 1 M magnesium chloride, brought to pH 8 with sodium hydroxide, was conducted to determine the exchangeable P fraction. Afterwards, Fe-bound P was targeted by extraction with a solution of 0.3 M trisodium citrate and 25 g/L sodium dithionite buffered to pH ∼7.6 in 1 M sodium bicarbonate (CDB solution) (8 h), followed by a wash step with 1 M MgCl2 for 0.5 hours. To extract authigenic Ca-P, the subsample residue was then extracted with 1 M sodium acetate buffered to pH 4 with acetic acid (6 h), again followed by a 1 M MgCl2 wash step for 0.5 hours. Detrital P was extracted with 1 M HCl for 24 hours. Finally, organic P was targeted with a 1 M HCl extraction for 24 hours after combusting the subsample residue at 550°C for 2 hours. The P concentrations in the CDB extracts were determined by ICP-OES, and the P concentrations in all other extracts were determined colorimetrically on a Shimadzu spectrophotometer with the molybdenum blue method [28]. All extractions were performed at room temperature and relative errors, calculated based on duplicates and in-house standards, were generally less than 10%. The first two steps of the SEDEX method for the anoxically preserved samples from stations 5, 8 and 10 were conducted in a N2-purged glove box to prevent sample oxidation and associated changes in P fractionation [11]. The sum of the sequential P fractions over the whole cores was similar to the total P concentrations as derived from the ICP-OES (<5% difference for all stations).

### Sediment iron fractionation

The sequential chemical Fe extraction method developed by Poulton and Canfield [29] was applied to subsamples of 0.1 g of sediment. First, an extraction with 1 M sodium acetate brought to pH 4.5 with acetic acid (24 h) targeted carbonate-associated Fe (Fe-Carb). Afterwards, the extraction with 1 M hydroxylamine-HCl in 25% v:v acetic acid (48 h) was used to extract the amorphous Fe-oxide fraction (Fe-Ox1). The crystalline Fe-oxides were then targeted with a 50 g/L sodium dithionite solution buffered to pH 4.8 with 0.35 M acetic acid/0.2 M sodium citrate for 2 hours (Fe-Ox2). Finally, magnetite was extracted with 0.2 M ammonium oxalate/0.17 M oxalic acid for 2 hours (Fe-Mag). All extractions were performed at room temperature. The extracts from stations 7614, 7617 and 7620 were measured with atomic absorption spectroscopy, and the samples from stations 5, 8 and 10 were measured with ICP-OES after dilution with 1 M HCl. The latter samples were extracted in a N2-purged environment to avoid sample oxidation and changes in associated Fe fractions [11]. Based on replicates, relative errors were generally less than 5%. The extraction method was also applied to freeze-dried and ground vivianite subsamples to study whether vivianite is dissolved in one of the Fe extraction steps. Vivianite was synthesized as described by [30] by mixing a deoxygenated solution of ammonium Fe(II) sulfate (12 g in 150 ml) with another deoxygenated 150 ml solution containing ammonium acetate (2 g) and disodium phosphate (10 g) under an inert argon (Ar) atmosphere. After two days the precipitate was filtered and freeze-dried. XRD-analysis confirmed that the synthesized mineral consisted of pure vivianite. We found that 95% of all vivianite was dissolved in the hydroxylamine-HCl and dithionite steps (0.2 g vivianite subsample in 10 ml solutions; 46 and 49% of total extracted P, respectively).

## Results

### Pore water

**Figure 2:**
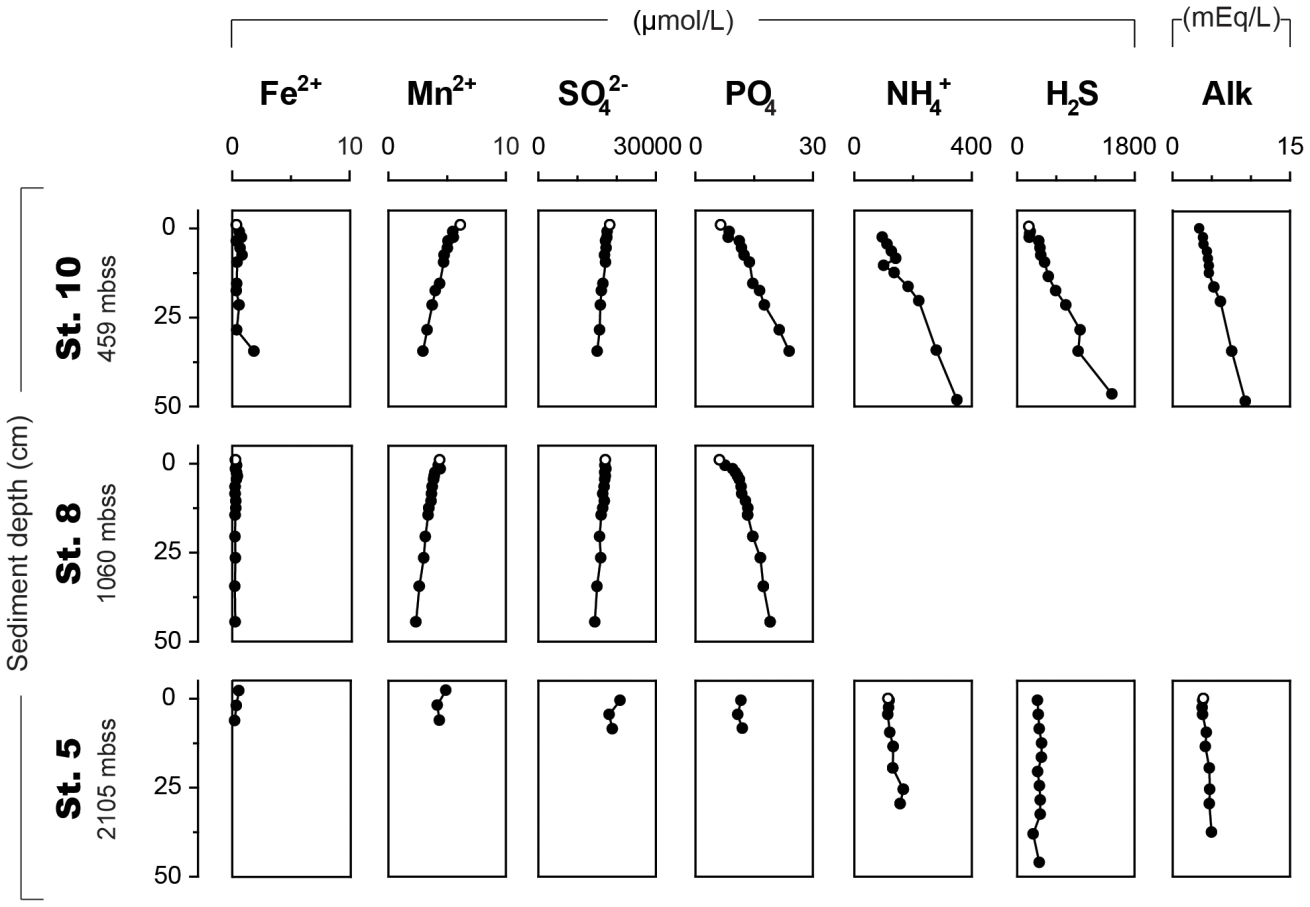
Pore water profiles of Fe^2+^, Mn^2+^, SO_4_^2−^, PO_4_, NH_4_^+^, H_2_S and alkalinity for three stations. Note the differences in depth resolution. Water depth is given in meters below sea surface (mbss). Bottom water samples are indicated with open symbols.

Pore water Fe^2+^ concentrations at stations 5, 8 and 10 are low (<2 µmol/L; Fig. 2; Dataset S1). Dissolved Mn^2+^ and SO_4_^2−^ both decrease with sediment depth, whereas PO_4_ increases with depth at all stations. At station 10, the NH_4_^+^, H_2_S and alkalinity profiles show a distinct increase with sediment depth to values of 380 µmol/L, 1400 µmol/L and 12 mEq/L, respectively. These trends are similar but less pronounced at station 5.

### Total sediment composition

**Figure 3:**
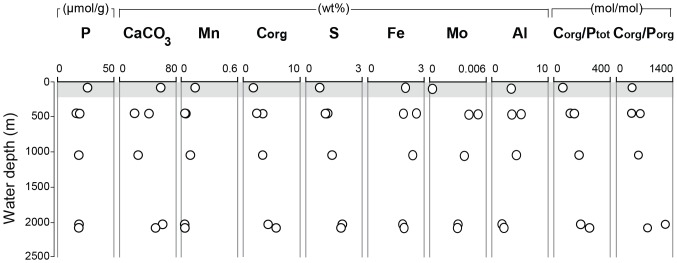
Trends with water depth for key characteristics of the sediment. Values are averages for the total analyzed Unit I section per multicore. The grey area indicates the part of the water column that is oxic.

Distinct changes in the geochemical composition of the sediment per site are observed upon the transition from the oxic shelf to the anoxic basin (Fig. 3; Dataset S2; whole core average, only Unit I). Sediments from the oxic shelf site 7614 are enriched in P, CaCO_3_ and Mn relative to sediments from the other sites that are located at greater depth in the basin (Fig. 3). These basin sediments are relatively enriched in organic C, S and Mo. Sediment Fe is more or less constant along the shelf-to-basin transects while Al is lowest at the two deepest stations. The ratios of organic C and total P (C_org_/P_tot_) and organic C and organic P (C_org_/P_org_) are lowest in the sediments underlying oxic bottom waters (station 7614).

**Figure 4:**
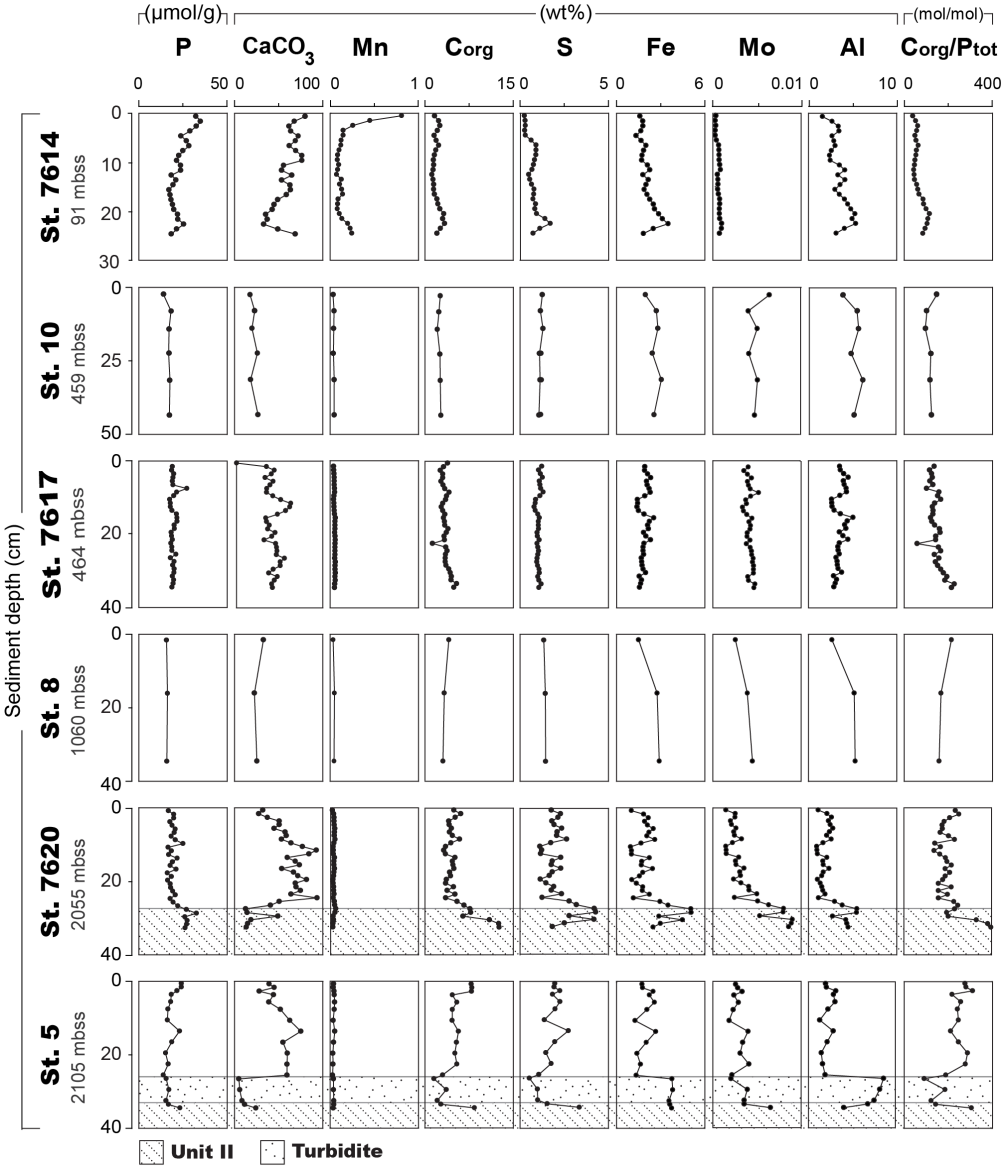
Sediment profiles of total P, CaCO_3_, Mn, C_org_ (organic C), S, Fe, Mo, Al and C_org_/P_tot_. The sediments at stations 10, 7617, 8, 7620 and 5 are Unit I sediments unless indicated otherwise. Note the difference in sampling resolution. Water depth is given in meters below sea surface (mbss).

Besides trends with water depth, distinct trends in chemical composition with depth in the sediment are observed. At the oxic station 7614, there is a strong enrichment in P, CaCO_3_ and Mn just below the sediment-water interface (Fig. 4). Total S, Fe, Al and C_org_/P_tot_ increase slightly with depth in the sediment, whereas Mo is low throughout the core. At most deeper sites, sediment P, CaCO_3_ and Mn are relatively constant with depth in Unit I. Only at the two deepest sites, CaCO_3_ profiles show an increase with sediment depth, followed by more constant values. Apart from a downcore increase at station 7620, no distinct trends in organic C and S are observed in the Unit I sediments. Trends in Fe, Mo and Al are roughly similar to those in S at most deep basin sites. There are significant geochemical changes around the Unit I/Unit II transition at station 7620. Here, CaCO_3_ decreases and organic C, Mo and C_org_/P_tot_ increase. The turbidite at station 5 also has a distinct geochemical signature with low CaCO_3_, organic C, S and C_org_/P_tot_ ratios and high Fe and Al, relative to unit I sediments.

### Sediment phosphorus and iron speciation

**Figure 5:**
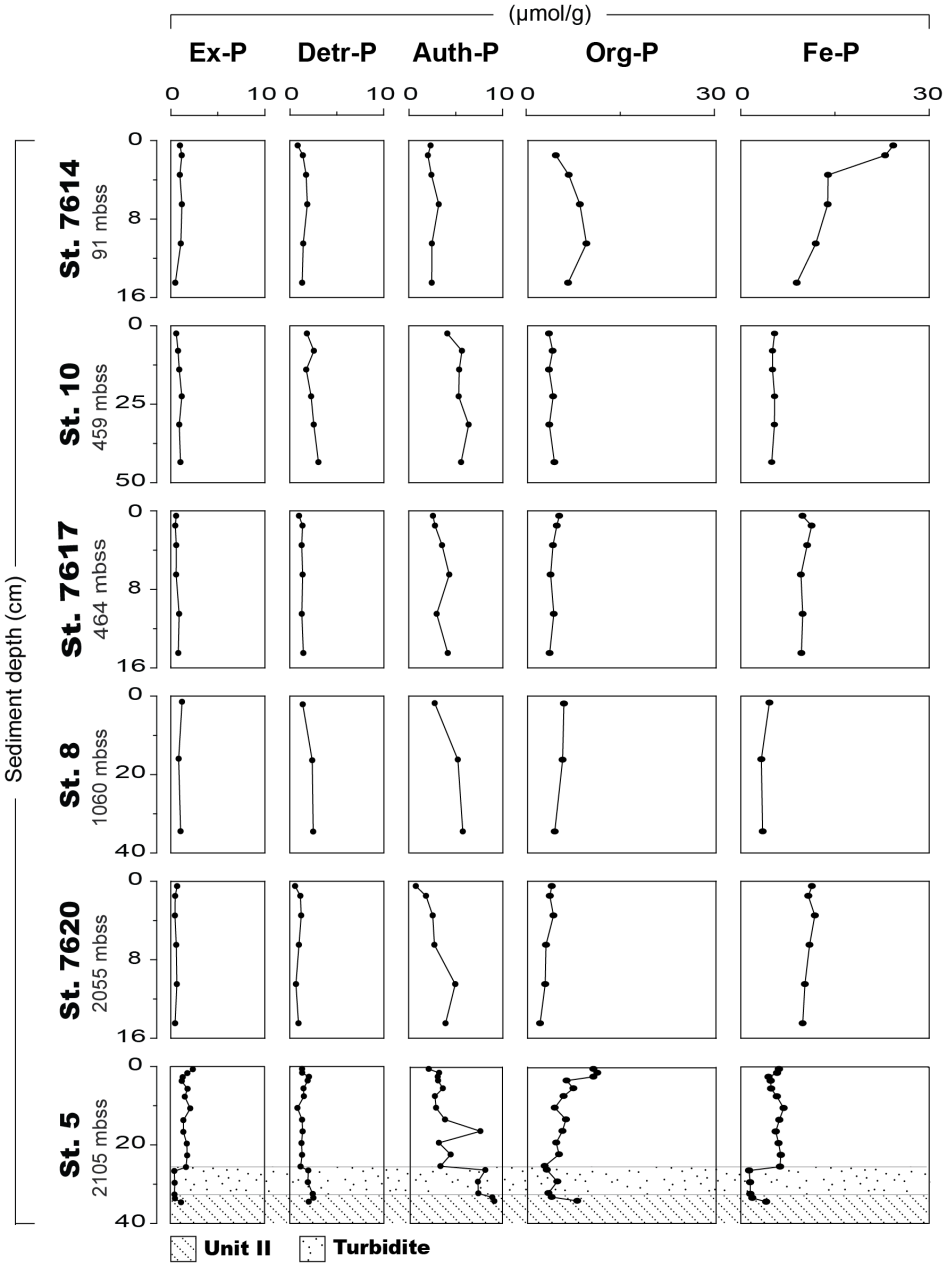
Solid phase phosphorus fractions for all stations (in µmol/g). The fractions are exchangeable P (Ex-P), detrital P (Detr-P), authigenic Ca-P (Auth-P), Organic P (Org-P) and Fe-bound P (Fe-P), as determined with the SEDEX extraction procedure [10]. All sediments were Unit I sediments except the turbidite and Unit II sediments at station 5. Note the differences in depth scale for each station. Water depth is given in meters below the sea surface (mbss).

At all stations, exchangeable and detrital P are low (<3 µmol/g) and account for only a minor proportion of total sediment P (each ∼10% of the total P pool) (Fig. 5; Dataset S3). The sediment from the oxic station 7614 contains some authigenic Ca-P and is enriched in organic P, relative to sediments from all deep basin stations except station 5. The major P fraction at station 7614 is Fe-bound P, which is enriched in the top 2 cm (∼24 µmol/g). At all anoxic stations, authigenic Ca-P generally increases with sediment depth, while organic P decreases slightly with sediment depth. Sediment samples from anoxic stations that were exposed to atmospheric oxygen during sample handling (station 7617 and 7620) have relatively high Fe-bound P concentrations and lower concentrations of all other sediment P forms, compared to samples from stations 5 and 10 which were kept strictly anoxic. The turbidite at station 5 is enriched in authigenic Ca-P and low in organic and Fe-bound P, relative to the overlying unit I deposits.

**Figure 6:**
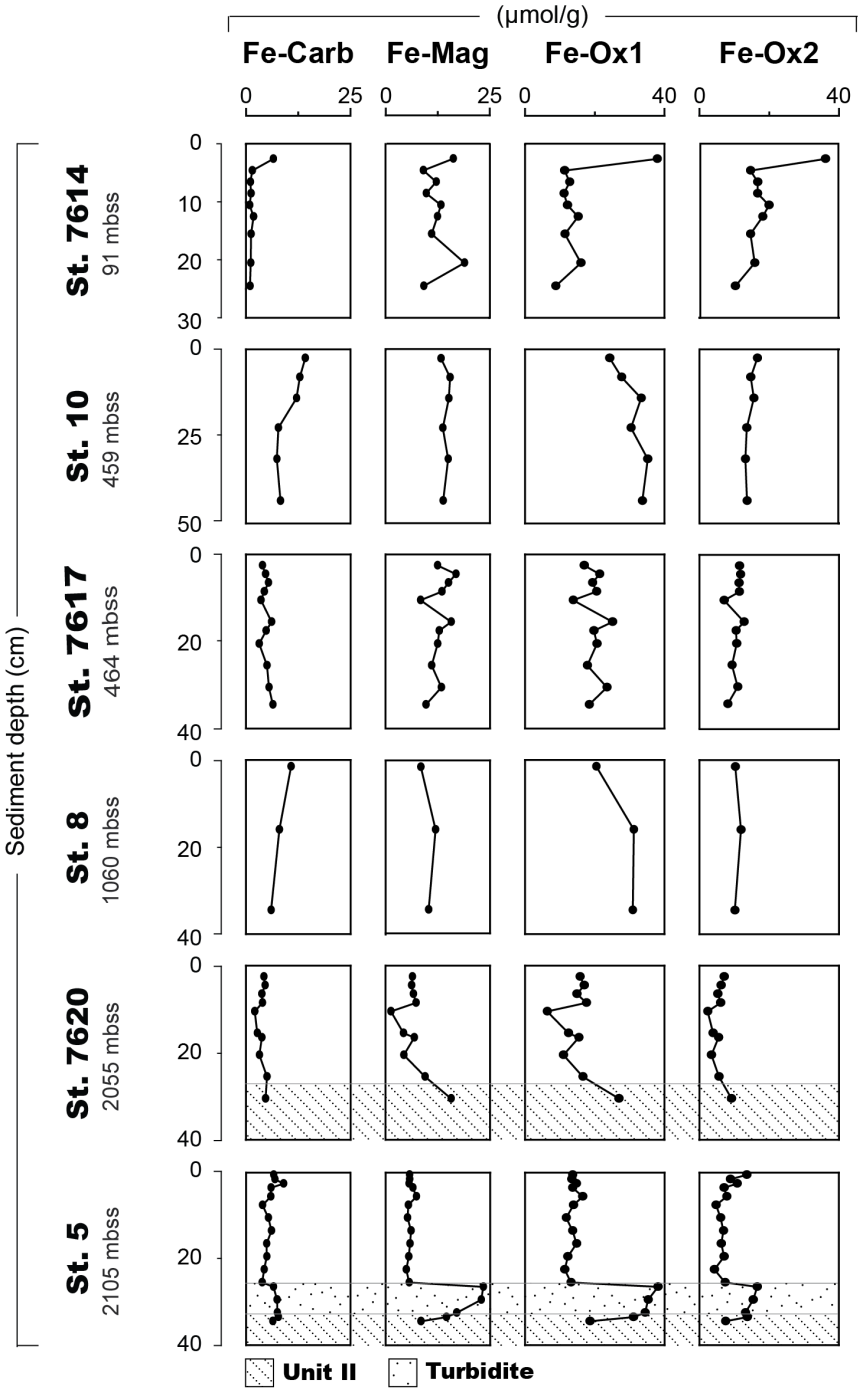
Solid phase iron fractions for all stations (in µmol/g). The fractions, as determined with the sequential extraction procedure of Poulton and Canfield [29], are carbonate-associated Fe (Fe-Carb) magnetite (Fe-Mag), Fe extracted with a hydroxylamine-HCl extraction (Fe-Ox1, targets amorphous Fe-oxides) and Fe extracted in the dithionite extraction (Fe-Ox2, targets crystalline Fe-oxides). All sediments were Unit I sediments except for the Unit II sediments at station 7620 and the turbidite and Unit II sediments at station 5. Note the differences in depth scale for each station. Water depth is given in meters below sea surface (mbss).

At all stations, the sum of the sequentially extracted Fe accounts for about 15% of total Fe. All extracted Fe fractions, but in particular the Fe-oxides (Fe-Ox1 and Fe-Ox2), are enriched in the oxic surface sediment at station 7614 and decrease within the upper 5 cm of sediment (Fig. 6; Dataset S4). Deeper in the sediment, the carbonate-associated Fe fraction is absent, while the other extracted Fe fractions (Fe-Mag, Fe-Ox1 and Fe-Ox2) are almost constant with values around 12, 15 and 17 µmol/g, respectively. In the Unit I sediments from the deep basin cores no clear trends in the extracted Fe fractions with sediment depth are observed. In these cores, the Fe fraction extracted in the hydroxylamine step (Fe-Ox1) generally is the major extracted Fe fraction. The turbidite at station 5 is enriched in all extracted Fe fractions, but particularly in Fe extracted in the oxalate and hydroxylamine steps (Fe-mag and Fe-ox1). Both Unit II and turbidite sediments are generally enriched in the extracted Fe fractions relative to Unit I.

## Discussion

### Fe-bound P burial in the deep basin sediments of the Black Sea

The near constant organic C, NH_4_^+^ and alkalinity profiles at the deepest station (Fig. 2 and 4) are in line with low rates of organic matter degradation in the anoxic sediments [31]. Also both C_org_/P_tot_ and C_org_/P_org_ in the basin sediments are elevated relative to the Redfield ratio of 106∶1 (Fig. 3; [32]) which is the average ratio for marine organic matter. This suggests preferential release of P relative to C from organic matter [33], [34], as typically is observed in sediments deposited under anoxic bottom waters [35]–[37]. Precipitation of the released P probably leads to formation of authigenic Ca-P in the basin sediments, and thus to a sink-switching of organic P to authigenic Ca-P [2], [5].

Our sequential P extraction data suggest remarkably high Fe-bound P contents in sediments from the deep basin of the Black Sea (Fig. 5). The burial of Fe-bound P in the basin sediments in the Black Sea occurs under sulfidic bottom waters (with 10 to 400 µmol H_2_S/L, depending on water depth [16]). These reducing conditions in the basin sediments are also reflected by enrichments in sedimentary S and Mo, which both precipitate under sulfidic conditions. In contrast, the surface sediments underlying oxic bottom water at station 7614 are enriched in Mn (Fig. 3). The combined results confirm that Fe-bound P is an important burial sink for P in anoxic basin sediments, as shown recently for the Baltic Sea [9].

### Alterations in P and Fe fractionation due to oxidation artifacts

Because some of the investigated sediments were exposed to the atmosphere during sample handling (stations 7614, 7617, 7620), there is the possibility that the original P fractionation was altered and that Fe-bound P was formed as an oxidation artifact. In these sediments, sulfuric acid, as produced by the oxidation of FeS_x_, can lead to the dissolution of authigenic and detrital Ca-P. Exchangeable and organic P may also decline upon oxic storage. The released PO_4_ can then be bound to freshly formed Fe-oxides, which results in a higher Fe-bound P fraction in the sediment [11], [38].

This may explain the higher Fe-bound P fractions in the sediments that were exposed to the atmosphere. If so, this implies that the conversion to Fe-bound P can also occur in CaCO_3_ rich sediments (∼50 wt%; Fig. 3), despite the buffering capacity of CaCO_3_ against acidic dissolution of authigenic Ca-P. Nevertheless, oxidation artifacts alone cannot explain the presence of Fe-bound P in the Black Sea sediments given that the sediments that were analyzed under strictly anoxic conditions also contain Fe-bound P (Fig. 5).

### Are turbidites or Fe from the water column possible sources of Fe-bound P in the deep basin sediments?

The Fe-bound P in the basin sediments is determined with the CDB-step of the SEDEX procedure, which targets the Fe-oxide bound P fraction in the sediment [10]. This requires the presence of Fe (oxyhydr)oxides in the basin sediments. The shelf-to-basin Fe shuttle [39]–[41] can lead to Fe enrichments in the basin, which is reflected in our data by the increasing sedimentary Fe/Al ratio with water depth (Fig. 3). However, the excess Fe in the basin is most likely deposited as Fe sulfides. In the sulfidic water column, H_2_S reacts rapidly with Fe (oxyhydr)oxides (on time scales of seconds to weeks), while the travel time of Fe from the shelf sediments to the basin sediments is estimated to lie around 1500 days [41], [42]. It is also unlikely that the Fe (oxyhydr)oxides are transported via turbidites at all basin locations, as we could only identify a turbidite at station 5 while all basin sediments were enriched in Fe-bound P. It is thus highly unlikely that the Fe-bound P in the anoxic basin sediments represents Fe-oxide bound P.

### Vivianite as a possible Fe-bound P phase in the deep basin sediments

Still, in all Unit I sediments, large amounts of Fe were extracted in the hydroxylamine-HCl and dithionite steps (Fig. 6), which together represent the pool of sedimentary Fe (oxyhydr)oxides [29]. It is possible that some labile FeS_x_ was extracted during treatment with either hydroxylamine-HCl or dithionite. Another possibility is that the extracted Fe was originally present in reduced Fe phosphates such as vivianite (Fe(II)_3_(PO_4_)_2_). As vivianite is also dissolved in the CDB- step of the sequential P extractions [12], the relatively large amounts of Fe-bound P as well as Fe extracted in the hydroxylamine-HCl and dithionite step in strongly reducing Unit I sediments in the Black Sea may in fact reflect the presence of vivianite.

The lack of a change of the Fe-bound P with sediment depth in the sediments overlain by an anoxic water column suggests the presence of a stable Fe phosphate mineral (Fig. 5). In contrast, Fe-bound P in the sediments that are overlain by an oxic water column sharply decreases in the upper 3–5 cm of the sediment, due to reductive dissolution of Fe (oxyhydr)oxides (Fig. 5). A parallel redox-sensitive dissolution of Mn-oxides is observed at this station as well (Fig. 4). Note that, besides vivianite, also other reduced Fe phosphates as ludlamite Fe_3_(PO_4_)_2_.4(H_2_O) and phosphoferrite Fe_3_(PO_4_)_2_.3(H_2_O) [43] might be dissolved in the P and Fe extraction steps and may explain the high Fe-bound P fractions at the anoxic basin sites.

### Inclusion of Fe-bound P in Deltaproteobacteria

The formation of vivianite is assumed to be minimal in euxinic environments due to efficient scavenging of dissolved Fe^2+^ by H_2_S [42]. However, processes in micro-environments in the sediments or overlying water column, which are not controlled by the pore water geochemistry, may still allow vivianite formation, as suggested by Jilbert and Slomp [9]. These microenvironments could be established in *Deltaproteobacteria*.

*Deltaproteobacteria* that are closely related to known sulfate-reducers and zero-valent sulfur (S^0^) disproportionators were recently observed in bacterial mats performing anaerobic oxidation of methane in the anoxic Black Sea [44]–[46]. Raman spectroscopy indicated that these bacteria contain vivianite granules [46]. Iron- and P- rich inclusions in *Deltaproteobacteria* have also been observed in a bacterial culture from the Mediterranean Sea [47]. The function of these inclusions is so far unknown. However, it has been suggested that similar inclusions are involved in the detoxification of Fe [48] or magnetotaxis [49].

Such Fe- and P-rich inclusions in *Deltaproteobacteria* bacteria may explain the occurrence of Fe-bound P in the anoxic basin sediments as observed in this study. Note that the Fe-rich turbidite, which probably originated from the Black Sea shelf, does not contain significant Fe-bound P. This suggests that the reduced Fe-bound P phases are not formed in surface sediments on the shelf. This is in line with observations in the Baltic Sea showing that Fe-bound P is completely lost at depth in the sediment at oxic and hypoxic sites [6], [9].

The P accumulation in bacteria may also play an important role in sedimentary P burial in other marine environments. *Deltaproteobacteria* are abundantly present in and around the SMTZ in many marine systems (e.g. [50]). Fe-P inclusions in these bacteria may help explain the enrichment in Fe-bound P observed around the STMZ in Baltic Sea sediments [9]. For other bacteria it is already known that they can affect sedimentary P burial. It has, for instance, been shown that sulfide-oxidizing bacteria on the Nambian shelf can induce precipitation of authigenic Ca-P in the sediment (>15 mmol/g; [51], [52]).

### Fe-bound P burial in the basin sediments as a product of the Mn-Fe-P shuttle

Micro-environments in sinking material in the water column may already facilitate the formation of stable Fe-bound P phases. Dellwig et al. [53] observed almost pure Fe-P particles within and below the redoxcline of the Black Sea. The formation of nearly pure Fe-P phases has been found to occur as the result of reductive dissolution of Mn-oxides from mixed Mn-Fe-P phases (the so-called Mn-Fe-P shuttle). Based on an average molar Fe/P ratio of 2.9 for these Fe-P particles, Dellwig et al. [53] argued that they are unstable colloidal P-bearing hydrous ferric oxides rather than stable Fe phosphates such as vivianite or strengite. They assumed that the Fe-P particles are dissolved in the sulfidic part of the Black Sea water column below the redoxcline. However, the exact Fe-P phase and their fate in sulfidic waters are unknown. If some Fe-P particles survive the passage through the sulfidic water column, perhaps through alteration to stable Fe phosphates, the Mn-Fe-P-shuttle may contribute to Fe-bound P deposition and thus Fe-bound P burial in the underlying basin sediments.

### A simple P budget for the Black Sea

Here, we attempt to assess the importance of Fe-bound P burial for the P cycle in the Black Sea using a simple mass budget (Table 2). The Black Sea is divided into two areas: the shelf (0–200 m water depth) and the basin (>200 m water depth). The P input consists of river input, deposition of atmospheric dust and inflow from the Bosporus. Some P is removed via outflow through the Bosporus but the main sink is sedimentary P burial. To obtain P accumulation rates (in 10^7^ mol/yr), mass accumulation rates per sediment surface area (g/m^2^/yr) derived from Teodoru et al. [35] were multiplied by the total sediment surface area (m^2^) and the P content as derived from our sequential P extraction data (mol/g; Fig. 5). In the sediments overlain by oxic bottom water, the top 5 cm of sediment was probably enriched in Fe-oxide bound P, of which most is reductively dissolved in the deeper sediment (Fig. 5). To focus on long-term P burial, the top 5 cm from the shelf sediment was excluded in the calculations of the mean P content and relative P fractions. Furthermore, the P fractionation data from stations 7617 and 7620 were excluded as the P fractions might be altered by oxidation artifacts [11].

**Table 2:** Sources of data for the P budget for the Black Sea in x 10^7^ mol/yr, unless noted differently.

	Shelf (0–200 m)	Basin (>200 m)	Source
Total sediment surface area (km^2^)	145 500	267 500	[35] and references therein
Mean mass accumulation rate (g/m^2^/yr)	264	63	[35] and references therein
Mean P content (µmol/g)	24.5	18.0	Own data from SEDEX (*)
Calculated P accumulation rate	94	30	
P input by rivers	142 for period 1996–2000		[35]
Atmospheric P input	10		[57], [58]
Bosporus P input	32		[59]
Bosporus P output	49		[59]

Mean values for the relative P fractions were calculated from the P fractionation data (Fig 5).

*: based on sediments from 5 cm sediment depth downwards.

**Figure 7:**
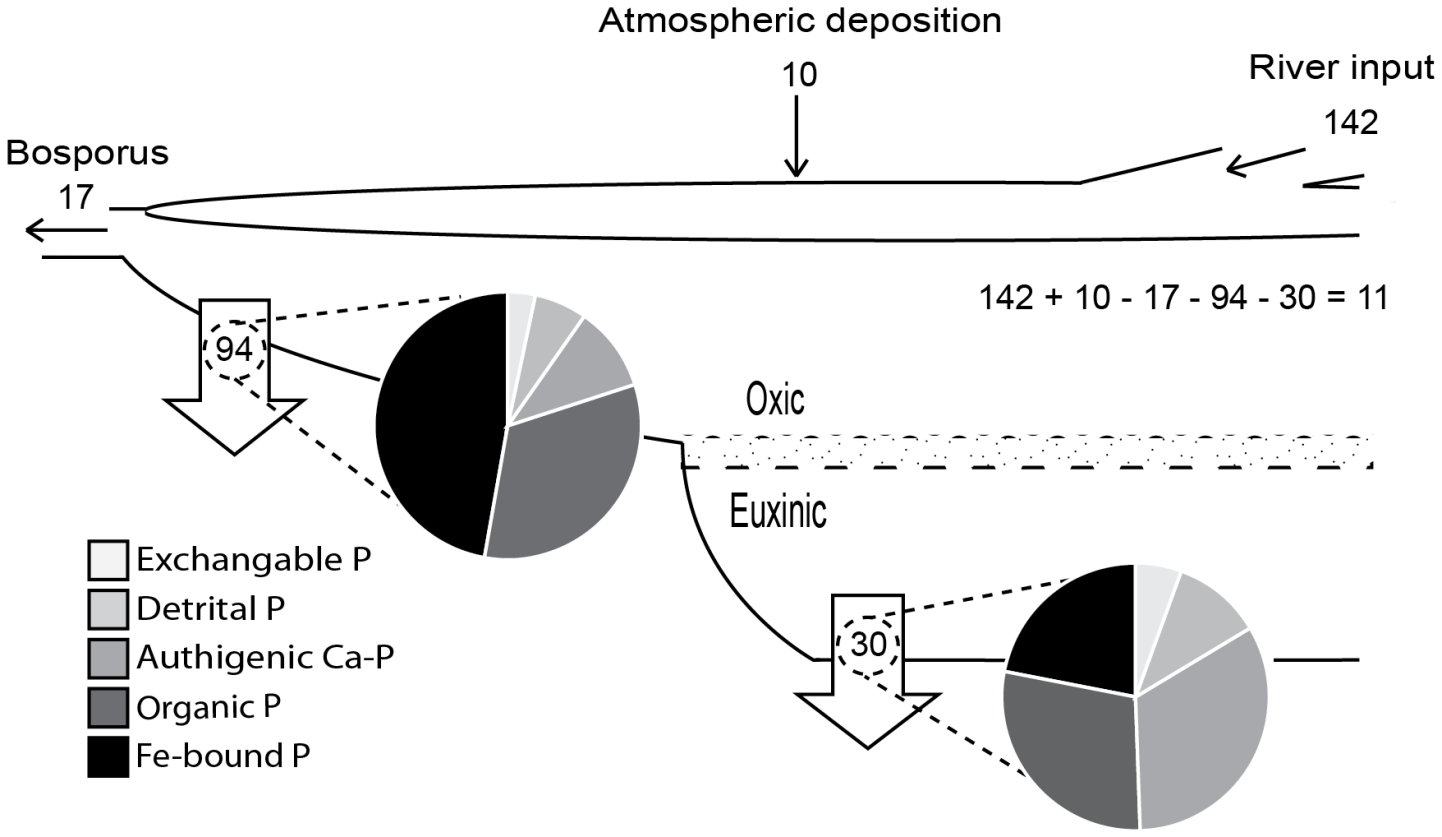
The P mass balance of the Black Sea with values in x 10^7^ mol P/yr. The fluxes related to the Sea of Marmara are excluded as their effect is minimal [35]. The Black Sea is divided into two areas: the shelf with generally oxic bottom waters (<200 m water depth) and the basin with anoxic bottom waters (>200 m water depth). The relative P fractions are based on values below 5 cm depth to limit the effect of short-term burial on total P burial. Based on this balance, 11×10^7^ mol P is added to the water column on a yearly basis.

Our budget clearly shows that P burial is the most important removal pathway for P in the Black sea (Fig. 7), as also shown by Arthur and Dean [18] and Teodoru et al. [35]. Without any P burial, the residence time of PO_4_ in the water column would increase 8-fold, from approximately 2000 years to 16000 years (based on recent mean PO_4_ concentrations throughout the water column (∼5 µmol/L [53]) and a water volume of 547000 km^3^ [54]). With our calculated P burial rates, 11×10^7^ mol P/yr would be added to the water column, which would result in less than a 5% increase in PO_4_ concentrations in 1000 years. However, without any P burial, PO_4_ concentrations would increase by approximately 50% in 1000 years. With higher yearly P river inputs (e.g. 220×10^7^ mol P/yr; [55] and references therein) and no P burial, PO_4_ concentrations in the water column would increase by 4 µmol/L in 1000 years.

As observed in many marine systems [4], more burial of P occurs on the shelf than in the deep basin (Fig. 7). The calculated P burial on the shelf is 94×10^7^ mol P/yr, whereas the calculated burial of P in the deep basin is 30×10^7^ mol P/yr. The actual P burial on the shelf might even be higher as shelf areas with high P accumulation rates were not included. Teodoru et al. [35] who did include sites from the Donau and Dniestr delta front, calculated higher burial rates of 116×10^7^ mol/yr on the shelf. Note that it is, however, unknown to what extent P burial on the shelf contributes to long-term P burial. Most P is buried as Fe-bound P and further work is required to determine whether this P is truly buried permanently as Fe-bound P or undergoes sink-switching to authigenic Ca-P [2], [5].

Based on our calculations, at least 30% of the total P burial occurs in the deep basin of the Black Sea, where Fe-bound P burial is nearly as important as authigenic and organic P burial (22%, 32% and 29%, respectively). The latter results are in correspondence with results from an anoxic basin in the Baltic Sea (site F80), where Fe-bound P, authigenic P and organic P were also the main sedimentary P phases (29%, 21% and 36% of the total extracted P from 5 cm sediment depth downwards, respectively) [9]. These estimates show that Fe-bound P burial is more important in anoxic, sulfidic basins than currently assumed. We find that burial of Fe-bound P lowers C_org_/P_tot_ ratios in sediments of the Black Sea basin by 22%. The burial of Fe-bound P thus reduces the magnitude of the enhanced regeneration of P relative to organic C that is typically observed under anoxic, sulfidic conditions [56].

## Conclusions

Our results show that Fe-bound P is an important P fraction in anoxic basin sediments in the Black Sea, accounting for more than 20% of the total sediment pool of P. The burial of Fe-bound P is of sufficient magnitude to impact the P cycle in the water column of the Black Sea. Together with recent observations of high Fe-bound P burial in the Baltic Sea, our results suggest that the burial of Fe-bound P in anoxic basins is more common than typically assumed. Several mechanisms may perhaps lead to the burial of Fe-bound P in the Black Sea basin. One possibility is that this P is present in the form of reduced Fe-P minerals that might be formed by sulfur-disproportionating *Deltaproteobacteria*. Further research is needed to elucidate the exact mineral form and the mechanisms that lead to the burial of Fe-bound P in anoxic basins.

## Supporting Information

Dataset S1 Pore water data.
(PDF)

Dataset S2 Solid phase data.
(PDF)

Dataset S3 Solid phase phosphorus fractions.
(PDF)

Dataset S4 Solid phase iron fractions.
(PDF)
