# Supplementary material for: Are Iron-Phosphate Minerals a Sink for Phosphorus in Anoxic Black Sea Sediments?
Source: PLoS One. 2014 Jul 2;9(7):e101139. doi: 10.1371/journal.pone.0101139 (PMC4079231; doi:10.1371/journal.pone.0101139)
Supplement: Dataset S1 — Pore water data. (PDF) [file pone.0101139.s001.pdf]

Dataset S1: Pore water data

**Station**	**Core**	**Sediment depth**	**Fe 2+**	**Mn 2+**	**SO4 2-**	**PO 4**	**H2S***	**Core**	**Sediment depth**	**NH4+**	**Alk**
		average (cm)	µmol/L	µmol/L	mmol/L	µmol/L	µmol/L		*average*	mmol/L	*mEq/L*
10	MC-10-04	-1.00	0.31	6.15	18.34	6.45		MC-10-05	0.00		3.42
10	MC-10-04	-1.00	0.31	6.09	18.08	6.38		MC-10-05	2.50	0.10	3.89
10	MC-10-04	Mean bottom water	0.31	6.12	18.21	6.41	182.30	MC-10-05	4.50	0.11	3.97
10	MC-10-04	0.90	0.55	5.48	17.51	8.59	201.81	MC-10-05	6.50	0.13	4.41
10	MC-10-04	1.50					193.56				
10	MC-10-04	2.50	0.73	5.53	17.49	8.36	186.80	MC-10-05	8.50	0.14	4.52
10	MC-10-04	3.50	0.30	5.07	17.13	11.21	333.84	MC-10-05	10.50	0.10	4.66
10	MC-10-04	5.50	0.62	5.02	17.27	11.76	351.85	MC-10-05	12.50	0.14	4.66
10	MC-10-04	7.50	0.79	4.74	16.88	12.43	363.10	MC-10-05	16.50	0.18	5.30
10	MC-10-04	9.50	0.36	4.70	17.16	13.80	422.37	MC-10-05	20.50	0.22	6.14
10	MC-10-04	13.50					479.39				
10	MC-10-04	15.50	0.33	4.35	16.49	14.64		MC-10-05	34.50	0.28	7.57
10	MC-10-04	17.50	0.29	3.99	16.05	16.36	591.92	MC-10-05	48.50	0.35	9.30
10	MC-10-04	21.50	0.53	3.73	15.82	17.58	746.46				
10	MC-10-04	28.50	0.32	3.29	15.59	21.42	967.78				
10	MC-10-04	34.50	1.80	2.93	15.01	23.94	937.77				
10	MC-10-04	46.50					1455.41				
8	MC-08-02	-1.00	0.11	4.35	17.06	6.14					
8	MC-08-02	0.50	0.19	4.25	16.97	7.54		***other core from the same deployment**			
8	MC-08-02	1.50	0.11	4.41	17.22	9.49					
8	MC-08-02	2.50	0.20	3.95	16.95	10.17					
8	MC-08-02	3.50	0.27	3.89	17.08	10.76					
8	MC-08-02	4.50	0.17	3.85	16.95	11.19					
8	MC-08-02	6.50	0.08	3.73	16.79	11.67					
8	MC-08-02	8.50	0.08	3.67	16.42	11.80					
8	MC-08-02	10.50	0.12	3.64	16.82	12.77					
8	MC-08-02	12.50	0.12	3.44	16.40	13.36					
8	MC-08-02	14.50	0.08	3.37	16.04	13.32					
8	MC-08-02	20.50	0.07	3.14	15.61	14.65					
8	MC-08-02	26.50	0.09	3.00	15.91	16.57					
8	MC-08-02	34.50	0.05	2.64	14.97	17.34					
8	MC-08-02	44.50	0.07	2.35	14.40	19.07					
5	MC-05-14	0.50	0.48	4.89	20.83	11.60	313.86	MC-05-03	0.00	0.12	3.95
5	MC-05-14	1.50						MC-05-03	0.50	0.12	3.87
5	MC-05-14	2.50						MC-05-03	2.50	0.12	3.80
5	MC-05-14	3.50						MC-05-03	4.50	0.11	3.85
5	MC-05-14	4.50	0.26	4.15	18.03	10.77	326.88	MC-05-03	9.50	0.12	4.34
5	MC-05-14	5.50						MC-05-03	13.50	0.13	4.21
5	MC-05-14	7.50						MC-05-03	19.50	0.13	4.71
5	MC-05-14	8.50	0.13	4.35	18.86	11.94	342.61	MC-05-03	25.50	0.17	4.76
5	MC-05-14	12.50					377.70	MC-05-03	29.50	0.16	4.71
5	MC-05-14	16.50					377.36	MC-05-03	37.50		4.98
5	MC-05-14	20.50					318.84				
5	MC-05-14	24.50					346.78				
5	MC-05-14	28.50					356.42				
5	MC-05-14	32.50					357.18				
5	MC-05-14	38.00					248.51				
5	MC-05-14	46.00					344.16				
