# Supplementary material for: Are Iron-Phosphate Minerals a Sink for Phosphorus in Anoxic Black Sea Sediments?
Source: PLoS One. 2014 Jul 2;9(7):e101139. doi: 10.1371/journal.pone.0101139 (PMC4079231; doi:10.1371/journal.pone.0101139)
Supplement: Dataset S2 — Solid phase data. (PDF) [file pone.0101139.s002.pdf]

Dataset S2: Solid phase data

Station	Core	Sediment depth			P	CaCO3	Mn	Corg	S	Al	Fe	Fe/Al	Mo	Mo/Al	Corg/Ptot
		top (cm)	*bottom (cm)*	*average (cm)*	µmol/g	*wt%*	*wt%*	*wt%*	*wt%*	*wt%*	*wt%*	*wt%/wt%*	*wt%*	*wt%/wt%*	*mol/mol*
10	MC-10-05	2	3	2.50	14.01	17.58	0.03	2.49	1.26	3.88	1.96	0.50	0.007	0.0018	147.92
10	MC-10-05	7	9	8.00	18.23	22.84	0.04	2.26	1.16	5.51	2.71	0.49	0.004	0.0008	103.30
10	MC-10-05	13	15	14.00	17.17	19.63	0.04	1.98	1.30	5.71	2.81	0.49	0.005	0.0009	96.17
10	MC-10-05	21	24	22.50	16.89	25.77	0.04	2.45	1.11	4.87	2.44	0.50	0.004	0.0009	120.82
10	MC-10-05	30	33	31.50	17.69	18.16	0.04	2.49	1.14	6.18	3.04	0.49	0.005	0.0009	117.32
10	MC-10-05	42	45	43.50	17.36	26.47	0.04	2.60	1.07	5.18	2.55	0.49	0.005	0.0010	124.72
8	MC-08-02	1	2	1.50	15.70	32.64	0.03	4.04	1.35	2.65	1.49	0.56	0.003	0.0010	214.14
8	MC-08-02	15	17	16.00	16.25	22.47	0.05	3.26	1.43	5.19	2.76	0.53	0.004	0.0008	166.92
8	MC-08-02	33	36	34.50	15.94	25.23	0.04	3.03	1.45	5.29	2.91	0.55	0.005	0.0009	158.40
5	MC-05-14	0	1	0.50	24.31	39.09	0.04	8.09	1.98	1.98	1.73	0.87	0.003	0.0013	276.97
5	MC-05-14	1	2	1.50	24.27	44.73	0.03	8.19	1.93	2.08	1.75	0.84	0.003	0.0014	280.93
5	MC-05-14	2	3	2.50	21.75	28.05	0.04	8.14	2.26	3.16	2.49	0.79	0.003	0.0011	311.57
5	MC-05-14	3	4	3.50	18.56	44.15	0.05	4.85	1.84	2.88	2.23	0.77	0.002	0.0009	217.76
5	MC-05-14	5	6	5.50	18.15	38.86	0.05	5.61	2.26	3.06	2.57	0.84	0.003	0.0010	257.28
5	MC-05-14	7	8	7.50	16.63	51.96	0.05	4.85	1.98	2.26	2.06	0.91	0.002	0.0011	242.68
5	MC-05-14	10	11	10.50	16.30	62.50	0.03	4.83	1.38	1.30	1.25	0.96	0.002	0.0015	246.76
5	MC-05-14	13	14	13.50	23.22	75.14	0.05	5.90	2.74	2.87	2.67	0.93	0.004	0.0014	211.58
5	MC-05-14	16	17	16.50	18.80	54.79	0.04	5.58	1.97	2.16	2.01	0.93	0.004	0.0016	247.00
5	MC-05-14	19	20	19.50	15.26	59.71	0.03	5.29	1.46	1.47	1.38	0.93	0.003	0.0022	288.49
5	MC-05-14	22	23	22.50	16.82	59.39	0.03	5.62	1.76	1.63	1.62	1.00	0.004	0.0026	278.22
5	MC-05-14	25	26	25.50	13.99	59.38	0.03	3.18	1.06	1.93	1.33	0.69	0.002	0.0012	189.04
5	MC-05-14	26	27	26.50	15.61	4.92	0.04	1.71	0.53	8.67	3.77	0.43	0.002	0.0002	91.09
5	MC-05-14	29	30	29.50	17.18	5.83	0.04	3.83	0.99	8.20	3.82	0.47	0.004	0.0005	185.63
5	MC-05-14	32	33	32.50	15.52	8.49	0.04	2.29	1.00	7.57	3.56	0.47	0.004	0.0005	122.65
5	MC-05-14	33	34	33.50	16.85	11.38	0.04	2.89	1.54	6.82	3.62	0.53	0.004	0.0005	143.04
5	MC-05-14	34	35	34.50	23.44	24.10	0.03	8.62	3.36	4.07	3.75	0.92	0.007	0.0017	306.14
7614	7614-1	0	1	0.50	32.54	80.40	0.81	1.44	0.25	1.53	1.47	0.96	0.000	0.0001	36.93
7614	7614-1	1	2	1.50	35.06	67.86	0.45	2.11	0.29	2.63	1.67	0.64	0.000	0.0000	50.10
7614	7614-1	2	3	2.50	32.78	61.75	0.25	2.36	0.30	3.35	1.68	0.50	0.000	0.0000	59.84
7614	7614-1	3	4	3.50	29.14	63.74	0.14	1.92	0.27	3.42	1.54	0.45	0.000	0.0000	54.73
7614	7614-1	4	5	4.50	23.97	72.35	0.14	1.37	0.30	2.62	1.20	0.46	0.000	0.0000	47.57
7614	7614-1	5	6	5.50	27.14	69.10	0.12	1.69	0.63	2.83	1.56	0.55	0.000	0.0000	51.95
7614	7614-1	6	7	6.50	28.61	62.04	0.11	2.12	0.91	2.98	1.89	0.64	0.000	0.0001	61.60
7614	7614-1	7	8	7.50	25.20	69.46	0.09	1.63	0.91	2.56	1.74	0.68	0.000	0.0002	53.84
7614	7614-1	8	9	8.50	22.77	76.50	0.08	1.41	0.90	2.34	1.62	0.69	0.000	0.0002	51.42
7614	7614-1	9	10	9.50	21.58	76.58	0.08	1.22	0.83	2.42	1.60	0.66	0.000	0.0002	47.03
7614	7614-1	10	11	10.50	23.81	55.67	0.09	1.29	0.77	3.48	1.99	0.57	0.001	0.0001	45.14
7614	7614-1	11	12	11.50	23.90	53.48	0.08	1.18	0.69	4.08	2.18	0.53	0.001	0.0001	41.03
7614	7614-1	12	13	12.50	18.57	65.01	0.07	0.96	0.50	3.30	1.68	0.51	0.000	0.0001	43.16
7614	7614-1	13	14	13.50	21.20	53.36	0.12	1.16	0.55	4.10	2.06	0.50	0.000	0.0001	45.58
7614	7614-1	14	15	14.50	19.56	63.00	0.10	1.29	0.66	3.55	1.90	0.54	0.000	0.0001	54.93
7614	7614-1	15	16	15.50	17.19	63.48	0.13	1.25	0.79	2.95	1.77	0.60	0.000	0.0001	60.53
7614	7614-1	16	17	16.50	17.89	58.75	0.14	1.41	0.78	3.46	1.97	0.57	0.000	0.0001	65.61
7614	7614-1	17	18	17.50	18.38	48.87	0.08	1.85	0.74	4.06	2.18	0.54	0.001	0.0001	83.90
7614	7614-1	18	19	18.50	19.24	44.77	0.08	2.00	0.89	4.41	2.43	0.55	0.001	0.0001	86.68
7614	7614-1	19	20	19.50	19.96	42.53	0.07	2.34	0.84	4.77	2.51	0.53	0.000	0.0001	97.68
7614	7614-1	20	21	20.50	22.10	34.97	0.10	3.02	0.93	5.24	2.79	0.53	0.000	0.0001	113.65
7614	7614-1	21	22	21.50	22.24	36.96	0.13	2.83	1.41	4.93	3.06	0.62	0.001	0.0001	106.08
7614	7614-1	22	23	22.50	25.61	32.85	0.19	3.16	1.72	5.32	3.45	0.65	0.001	0.0001	102.64
7614	7614-1	23	24	23.50	21.58	48.89	0.22	2.43	1.12	4.04	2.42	0.60	0.001	0.0002	93.81
7614	7614-1	24	25	24.50	18.51	68.94	0.24	1.84	0.74	3.09	1.71	0.55	0.000	0.0002	82.78
7617	7617	1	2	1.50	19.21	34.51	0.04	3.14	1.23	3.53	1.92	0.54	0.004	0.0011	136.01
7617	7617	2	3	2.50	18.74	43.71	0.04	2.60	1.05	3.60	1.90	0.53	0.004	0.0010	115.52
7617	7617	3	4	3.50	19.97	40.33	0.04	2.99	1.16	3.99	2.11	0.53	0.004	0.0010	124.55
7617	7617	4	5	4.50	19.50	33.13	0.05	3.00	1.27	4.51	2.34	0.52	0.004	0.0010	128.23
7617	7617	5	6	5.50	19.16	42.31	0.05	2.70	1.10	3.97	2.03	0.51	0.004	0.0010	117.37
7617	7617	6	7	6.50	19.44	38.74	0.04	3.04	1.13	4.05	2.09	0.52	0.004	0.0010	130.41
7617	7617	7	8	7.50	27.34	34.96	0.05	3.34	1.18	4.26	2.23	0.52	0.004	0.0010	101.62
7617	7617	8	9	8.50	21.56	34.78	0.05	4.08	1.31	4.33	2.30	0.53	0.005	0.0012	157.69
7617	7617	9	10	9.50	19.95	42.01	0.04	3.65	1.13	3.56	1.92	0.54	0.004	0.0012	152.31
7617	7617	10	11	10.50	17.55	51.10	0.04	3.50	0.88	2.63	1.41	0.54	0.004	0.0014	165.79
7617	7617	11	12	11.50	17.96	62.77	0.04	3.00	0.86	2.66	1.43	0.54	0.004	0.0015	139.21
7617	7617	12	13	12.50	17.95	61.56	0.04	2.76	0.80	2.68	1.39	0.52	0.003	0.0013	127.85
7617	7617	13	14	13.50	18.93	56.91	0.05	2.99	0.86	2.90	1.48	0.51	0.004	0.0012	131.41
7617	7617	14	15	14.50	21.50	47.38	0.05	3.37	1.00	3.63	1.87	0.51	0.004	0.0011	130.43
7617	7617	15	16	15.50	21.71	33.76	0.06	3.12	1.10	5.05	2.54	0.50	0.004	0.0009	119.78
7617	7617	16	17	16.50	21.83	34.83	0.05	3.30	1.03	4.40	2.24	0.51	0.004	0.0009	125.73
7617	7617	17	18	17.50	20.21	37.98	0.05	3.36	1.04	4.10	2.12	0.52	0.004	0.0010	138.48
7617	7617	18	19	18.50	20.26	36.12	0.05	3.90	1.04	4.25	2.18	0.51	0.004	0.0010	160.43
7617	7617	19	20	19.50	18.51	44.70	0.05	3.62	0.97	3.51	1.83	0.52	0.004	0.0011	163.00
7617	7617	20	21	20.50	18.65	40.74	0.05	3.22	0.99	3.91	1.99	0.51	0.004	0.0010	143.60
7617	7617	21	22	21.50	19.16	31.91	0.06	3.29	1.09	4.47	2.33	0.52	0.004	0.0010	142.87
7617	7617	22	23	22.50	18.21	45.37	0.06	1.28	0.96	3.51	1.86	0.53	0.004	0.0011	58.42
7617	7617	23	24	23.50	18.81	46.37	0.05	3.53	1.01	3.35	1.80	0.54	0.004	0.0013	156.06
7617	7617	24	25	24.50	18.87	46.81	0.05	3.76	1.05	3.37	1.80	0.53	0.004	0.0013	165.81
7617	7617	25	26	25.50	21.16	46.18	0.05	3.48	1.01	3.49	1.84	0.53	0.004	0.0013	136.88
7617	7617	26	27	26.50	18.07	55.53	0.05	3.43	0.97	3.12	1.69	0.54	0.005	0.0015	157.91
7617	7617	27	28	27.50	20.11	50.50	0.06	3.44	0.96	3.26	1.71	0.52	0.005	0.0014	142.21
7617	7617	28	29	28.50	19.38	50.70	0.06	3.55	1.00	3.27	1.74	0.53	0.005	0.0014	152.43
7617	7617	29	30	29.50	20.15	42.42	0.06	4.02	1.08	3.35	1.80	0.54	0.005	0.0014	166.04
7617	7617	30	31	30.50	19.99	37.31	0.06	4.28	1.10	3.76	1.97	0.52	0.005	0.0012	178.11

7617	7617	31	32	31.50	19.18	47.16	0.05	4.47	1.01	2.86	1.54	0.54	0.004	0.0014	194.18
7617	7617	32	33	32.50	19.87	43.83	0.06	4.42	1.07	3.19	1.70	0.53	0.004	0.0013	185.13
7617	7617	33	34	33.50	19.57	40.54	0.05	5.35	1.20	3.04	1.63	0.54	0.005	0.0016	227.48
7617	7617	34	35	34.50	18.92	41.70	0.05	4.88	1.07	2.88	1.56	0.54	0.005	0.0016	214.88
7620	7620	0	1	0.50	16.91	31.92	0.02	4.77	1.80	1.11	1.00	0.90	0.002	0.0014	235.10
7620	7620	1	2	1.50	19.86	27.36	0.03	5.98	2.33	2.11	1.83	0.87	0.003	0.0013	250.80
7620	7620	2	3	2.50	19.91	37.05	0.04	5.01	2.18	2.61	2.16	0.83	0.003	0.0010	209.39
7620	7620	3	4	3.50	17.75	50.56	0.04	3.92	1.84	2.27	1.90	0.84	0.002	0.0009	183.74
7620	7620	4	5	4.50	18.98	50.58	0.04	3.99	1.96	2.46	2.09	0.85	0.002	0.0009	175.15
7620	7620	5	6	5.50	20.72	44.80	0.05	4.39	2.40	2.77	2.50	0.90	0.003	0.0010	176.38
7620	7620	6	7	6.50	20.18	57.27	0.04	4.03	2.13	2.39	2.19	0.92	0.002	0.0010	166.30
7620	7620	7	8	7.50	18.45	58.59	0.04	4.52	2.11	2.07	2.05	0.99	0.003	0.0012	204.00
7620	7620	8	9	8.50	20.79	53.18	0.05	5.77	2.68	2.59	2.60	1.00	0.003	0.0013	231.14
7620	7620	9	10	9.50	25.12	63.98	0.04	4.27	1.77	1.67	1.66	1.00	0.002	0.0014	141.52
7620	7620	10	11	10.50	16.77	76.86	0.03	3.37	1.14	0.93	0.95	1.02	0.002	0.0016	167.36
7620	7620	11	12	11.50	18.59	92.65	0.03	3.07	1.25	0.97	1.01	1.04	0.002	0.0016	137.33
7620	7620	12	13	12.50	16.99	84.28	0.03	3.34	1.19	1.01	1.01	1.00	0.002	0.0016	163.69
7620	7620	13	14	13.50	21.83	59.68	0.04	4.98	2.35	2.09	2.22	1.06	0.003	0.0015	189.92
7620	7620	14	15	14.50	19.22	68.63	0.04	4.53	1.85	1.63	1.72	1.06	0.003	0.0017	196.29
7620	7620	15	16	15.50	17.80	73.54	0.04	4.64	1.80	1.67	1.69	1.01	0.003	0.0016	217.22
7620	7620	16	17	16.50	21.22	53.53	0.04	4.80	2.35	2.32	2.44	1.05	0.004	0.0016	188.31
7620	7620	17	18	17.50	16.24	66.88	0.04	4.08	1.85	1.67	1.77	1.06	0.003	0.0019	209.34
7620	7620	18	19	18.50	18.58	71.50	0.03	4.22	1.69	1.51	1.55	1.03	0.003	0.0022	189.25
7620	7620	19	20	19.50	16.42	81.50	0.03	3.51	1.16	1.02	1.03	1.01	0.002	0.0024	177.89
7620	7620	20	21	20.50	17.96	68.83	0.03	3.39	1.49	1.33	1.34	1.01	0.003	0.0025	157.33
7620	7620	21	22	21.50	18.22	69.75	0.03	4.74	1.94	1.52	1.78	1.17	0.004	0.0028	216.51
7620	7620	22	23	22.50	19.26	74.70	0.03	3.64	1.80	1.62	1.74	1.07	0.004	0.0027	157.35
7620	7620	23	24	23.50	20.80	63.87	0.04	4.99	2.39	1.89	2.22	1.18	0.005	0.0028	199.58
7620	7620	24	25	24.50	17.80	93.37	0.03	3.38	1.26	1.13	1.14	1.01	0.003	0.0023	157.94
7620	7620	25	26	25.50	19.50	50.56	0.04	5.34	2.83	2.95	2.95	1.00	0.005	0.0018	227.97
7620	7620	26	27	26.50	22.13	40.70	0.05	6.54	3.25	3.83	3.50	0.91	0.007	0.0017	246.11
7620	7620	27	28	27.50	27.07	12.62	0.06	7.55	4.27	5.47	5.05	0.92	0.008	0.0015	232.21
7620	7620	28	29	28.50	32.78	14.31	0.05	7.69	4.34	5.44	5.07	0.93	0.008	0.0015	195.26
7620	7620	29	30	29.50	26.15	49.14	0.03	6.37	2.83	2.74	2.87	1.05	0.006	0.0020	202.67
7620	7620	30	31	30.50	27.47	18.58	0.03	10.89	4.25	4.24	4.48	1.06	0.010	0.0022	330.16
7620	7620	31	32	31.50	27.14	14.92	0.03	12.48	2.56	4.36	2.98	0.68	0.009	0.0022	382.86
7620	7620	32	33	32.50	26.35	13.17	0.03	12.56	1.86	4.49	2.48	0.55	0.009	0.0020	396.81
