# Supplementary material for: Are Iron-Phosphate Minerals a Sink for Phosphorus in Anoxic Black Sea Sediments?
Source: PLoS One. 2014 Jul 2;9(7):e101139. doi: 10.1371/journal.pone.0101139 (PMC4079231; doi:10.1371/journal.pone.0101139)
Supplement: Dataset S3 — Solid phase phosphorus fractions. (PDF) [file pone.0101139.s003.pdf]

Dataset S3: Solid phase phosphorus fractions

Station	Core	Sediment depth			ex-P	Fe-P	authi-P	det-P	org-P
		top (cm)	*bottom (cm)*	*average (cm)*	µmol/g	µmol/g	µmol/g	µmol/g	µmol/g
10	MC-10-05	2	3	2.5	0.57	3.41	4.09	1.82	5.40
10	MC-10-05	7	9	8	0.77	3.99	5.64	2.56	5.06
10	MC-10-05	13	15	14	0.90	3.41	5.36	1.74	5.06
10	MC-10-05	21	24	22.5	1.17	4.07	5.30	2.27	5.40
10	MC-10-05	30	33	31.5	0.89	3.46	6.35	2.55	5.38
10	MC-10-05	42	45	43.5	1.03	4.27	5.56	3.05	4.94
8	MC-08-02	1	2	1.5	1.19	4.57	2.79	1.34	5.78
8	MC-08-02	15	17	16	0.83	3.28	5.28	2.36	5.58
8	MC-08-02	33	36	34.5	1.04	3.48	5.81	2.47	4.32
5	MC-05-14	0	1	0.5	2.33	6.12	2.01	1.27	10.47
5	MC-05-14	1	2	1.5	1.74	5.84	3.14	1.29	11.12
5	MC-05-14	2	3	2.5	1.24	4.45	2.98	2.00	10.48
5	MC-05-14	3	4	3.5	1.14	4.79	3.01	1.88	6.21
5	MC-05-14	5	6	5.5	1.76	4.88	3.54	1.43	7.31
5	MC-05-14	7	8	7.5	1.46	5.78	2.67	1.47	5.73
5	MC-05-14	10	11	10.5	2.05	6.88	2.80	0.79	4.32
5	MC-05-14	13	14	13.5	1.32	6.18	3.77	1.28	6.12
5	MC-05-14	16	17	16.5	1.32	5.59	7.61	1.33	5.55
5	MC-05-14	19	20	19.5	1.68	6.06	3.11	1.22	4.57
5	MC-05-14	22	23	22.5	1.72	6.42	4.37	1.27	5.01
5	MC-05-14	25	26	25.5	1.60	6.30	3.27	1.11	2.74
5	MC-05-14	26	27	26.5	0.33	1.32	8.13	1.93	3.05
5	MC-05-14	29	30	29.5	0.38	1.52	7.34	1.89	4.74
5	MC-05-14	32	33	32.5	0.36	1.53	7.38	2.40	3.33
5	MC-05-14	33	34	33.5	0.41	1.85	8.89	2.48	3.85
5	MC-05-14	34	35	34.5	1.06	4.09	9.12	1.99	7.92
7614	7614-1	0	1	0.5	0.97	24.30	2.12	0.84	0.00
7614	7614-1	1	2	1.5	1.16	22.99	1.82	1.39	4.45
7614	7614-1	3	4	3.5	0.96	13.91	2.21	1.73	6.51
7614	7614-1	6	7	6.5	1.17	13.86	3.01	1.86	8.29
7614	7614-1	10	11	10.5	1.06	11.95	2.26	1.43	9.33
7614	7614-1	14	15	14.5	0.48	8.93	2.25	1.31	6.41
7617	7617	0	1	0.5	0.57	9.85	2.57	0.98	5.03
7617	7617	1	2	1.5	0.48	11.28	2.79	1.35	4.66
7617	7617	3	4	3.5	0.58	10.57	3.54	1.26	4.04
7617	7617	6	7	6.5	0.56	9.60	4.30	1.37	3.68
7617	7617	10	11	10.5	0.88	9.88	2.97	1.28	4.17
7617	7617	14	15	14.5	0.80	9.67	4.15	1.44	3.51
7620	7620	0	1	0.5	0.65	11.34	0.75	0.56	3.84
7620	7620	1	2	1.5	0.45	10.76	1.84	1.13	3.54
7620	7620	3	4	3.5	0.43	11.81	2.55	1.21	4.13
7620	7620	6	7	6.5	0.57	10.97	2.73	0.97	2.94
7620	7620	10	11	10.5	0.63	10.22	4.94	0.65	2.81
7620	7620	14	15	14.5	0.44	9.88	3.91	0.92	2.00
