# Supplementary material for: Are Iron-Phosphate Minerals a Sink for Phosphorus in Anoxic Black Sea Sediments?
Source: PLoS One. 2014 Jul 2;9(7):e101139. doi: 10.1371/journal.pone.0101139 (PMC4079231; doi:10.1371/journal.pone.0101139)
Supplement: Dataset S4 — Solid phase iron fractions. (PDF) [file pone.0101139.s004.pdf]

Dataset S4: Solid phase iron fractions

Station	Core	Sediment depth			Fe-Carb	Fe-Ox1	Fe-Ox2	Fe-Mag
		top (cm)	*bottom (cm)*	*average (cm)*	µmol/g	µmol/g	µmol/g	µmol/g
10	MC-10-05	2.00	3.00	2.50	14.29	24.25	16.42	13.26
10	MC-10-05	7.00	9.00	8.00	12.95	27.65	14.55	15.43
10	MC-10-05	13.00	15.00	14.00	12.14	33.27	15.41	15.11
10	MC-10-05	21.00	24.00	22.50	7.80	30.34	13.40	13.68
10	MC-10-05	30.00	33.00	31.50	7.43	35.18	12.93	14.97
10	MC-10-05	42.00	45.00	43.50	8.32	33.58	13.43	13.77
8	MC-08-02	1.00	2.00	1.50	10.78	20.38	10.25	8.45
8	MC-08-02	15.00	17.00	16.00	7.98	31.13	11.91	11.94
8	MC-08-02	33.00	36.00	34.50	6.00	30.85	10.12	10.30
5	MC-05-14	0.00	1.00	0.50	6.61	13.56	13.54	5.67
5	MC-05-14	1.00	2.00	1.50	6.91	13.39	8.83	5.73
5	MC-05-14	2.00	3.00	2.50	9.04	14.66	10.75	5.57
5	MC-05-14	3.00	4.00	3.50	6.00	13.59	7.00	6.51
5	MC-05-14	5.00	6.00	5.50	5.94	16.50	7.80	7.36
5	MC-05-14	7.00	8.00	7.50	3.96	13.85	4.75	5.45
5	MC-05-14	10.00	11.00	10.50	5.34	11.73	6.03	5.21
5	MC-05-14	13.00	14.00	13.50	6.12	13.60	6.82	6.06
5	MC-05-14	16.00	17.00	16.50	4.93	14.71	6.19	5.81
5	MC-05-14	19.00	20.00	19.50	4.99	12.22	7.01	5.51
5	MC-05-14	22.00	23.00	22.50	4.35	11.37	4.25	4.95
5	MC-05-14	25.00	26.00	25.50	3.87	13.12	7.33	5.61
5	MC-05-14	26.00	27.00	26.50	6.56	38.07	16.51	23.33
5	MC-05-14	29.00	30.00	29.50	7.46	35.24	15.36	22.83
5	MC-05-14	32.00	33.00	32.50	7.52	34.33	13.16	17.00
5	MC-05-14	33.00	34.00	33.50	7.74	31.05	13.66	14.51
5	MC-05-14	34.00	35.00	34.50	6.40	18.57	7.46	8.46
7614	7614-1	2.00	3.00	2.50	6.51	37.84	36.20	16.29
7614	7614-1	4.00	5.00	4.50	1.50	11.31	14.63	9.13
7614	7614-1	6.00	7.00	6.50	1.04	12.73	16.72	12.21
7614	7614-1	8.00	9.00	8.50	1.19	11.14	16.61	9.79
7614	7614-1	10.00	11.00	10.50	0.86	12.15	19.96	13.34
7614	7614-1	15.00	16.00	15.50	1.21	11.42	14.59	11.10
7614	7614-1	20.00	21.00	20.50	1.13	16.00	15.79	19.01
7614	7614-1	24.00	25.00	24.50	0.97	8.73	10.26	9.21
7617	7617	2.00	3.00	2.50	3.91	16.96	11.46	12.41
7617	7617	4.00	5.00	4.50	4.67	21.32	11.74	16.83
7617	7617	6.00	7.00	6.50	5.36	19.33	11.32	15.03
7617	7617	8.00	9.00	8.50	4.36	20.46	11.44	13.49
7617	7617	10.00	11.00	10.50	3.57	13.77	7.08	8.34
7617	7617	15.00	16.00	15.50	6.13	25.05	12.79	15.68
7617	7617	20.00	21.00	20.50	3.16	20.63	10.66	12.46
7617	7617	25.00	26.00	25.50	5.00	17.82	9.31	11.04
7617	7617	30.00	31.00	30.50	5.46	23.42	10.93	13.35
7617	7617	34.00	35.00	34.50	6.43	18.37	8.08	9.67
7620	7620	2.00	3.00	2.50	4.26	15.75	7.11	6.42
7620	7620	4.00	5.00	4.50	4.56	16.94	6.19	6.24
7620	7620	6.00	7.00	6.50	3.78	14.88	5.27	6.66
7620	7620	8.00	9.00	8.50	3.90	17.54	6.03	7.28
7620	7620	10.00	11.00	10.50	2.08	6.35	2.32	1.31
7620	7620	15.00	16.00	15.50	2.69	12.43	3.91	4.31
7620	7620	20.00	21.00	20.50	3.21	10.98	3.41	4.37
7620	7620	25.00	26.00	25.50	5.01	16.56	5.58	9.46
7620	7620	30.00	31.00	30.50	4.69	26.91	9.19	15.66
